# Factors associated with pulmonary tuberculosis in LTBI individuals screened from close contacts: a retrospective case–control study

**DOI:** 10.3389/fpubh.2026.1804659

**Published:** 2026-07-02

**Authors:** Mengya He, Dingyong Sun, Weidong Wang, Xiaowan Xu, Chunyu Xie, Wenhui Xu, Wenjing Chang, Danwei Zheng, Yanqiu Zhang

**Affiliations:** Henan Center for Disease Control and Prevention, Zhengzhou, China

**Keywords:** associated factors, close contacts, latent tuberculosis infection, nomogram, pulmonary tuberculosis, tuberculosis

## Abstract

**Background:**

Latent tuberculosis infection (LTBI) progresses to pulmonary tuberculosis (PTB) in 5–10% of cases. This study aimed to identify factors associated with PTB among close contacts of PTB patients and to develop an exploratory nomogram for individualized risk estimation.

**Methods:**

A retrospective case–control study was conducted in Henan Province, China, in 2024. The PTB group comprised newly diagnosed PTB patients (*n* = 365), and the LTBI group comprised close contacts of PTB patients who tested positive on the purified protein derivative (PPD) test, with normal chest imaging and no clinical evidence of active TB after physician evaluation (*n* = 389). Participants were randomly divided into a training set (70%, *n* = 528) and a validation set (30%, *n* = 226). Multivariable logistic regression was performed to identify factors associated with PTB. An exploratory nomogram was developed based on the independent factors and internally validated using bootstrap resampling (1,000 replicates). Model performance was assessed using the area under the receiver operating characteristic (ROC) curve, calibration curves, and decision curve analysis (DCA).

**Results:**

Nine factors were independently associated with PTB. Factors associated with increased risk included age ≥60 years (OR = 2.261), male gender (OR = 3.587), diabetes (OR = 3.779), underweight (OR = 5.312), and self-reported high stress (OR = 1.862). Factors associated with decreased risk included married status (OR = 0.187), certain occupations (e.g., farmers, students), overweight (OR = 0.393), obesity (OR = 0.124), receipt of TB health education (OR = 0.446), and presence of a BCG scar (OR = 0.497). The exploratory nomogram showed AUCs of 0.842 (95% CI: 0.809–0.874) in the training set and 0.833 (95% CI: 0.780–0.885) in the validation set. Calibration curves demonstrated good agreement, and DCA suggested potential clinical utility.

**Conclusion:**

Age ≥60 years, male gender, diabetes, underweight, and high stress were associated with increased PTB risk, while married status, certain occupations, overweight, obesity, TB health education, and BCG scar were associated with decreased PTB risk among close contacts. The exploratory nomogram showed good discriminative ability in internal validation but requires external validation before clinical application.

## 1 Introduction

Tuberculosis (TB) is a chronic infectious disease caused by *Mycobacterium Tuberculosis* (MTB) (1). All organs can be affected, but the lungs are the most commonly affected (2). To this day, it remains one of the deadliest infectious diseases worldwide (2). The 2025 Global Tuberculosis Report indicates that there are an estimated 10.7 million new tuberculosis cases and approximately 1.23 million deaths worldwide in 2024 (3). Latent Tuberculosis Infection (LTBI) refers to the long-term immune response of the body to the antigen of MTB after infection, but there is no evidence in terms of clinical etiology or imaging (3). It is estimated that one quarter of the global population is infected with MTB, and the estimated number of LTBI individuals is about 2 billion (4). The number of LTBI individual in China is approximately 360 million, making it one of the countries with a high burden of LTBI in the world (5). 5 to 10% of LTBI cases can progress to active TB (6, 7). Individuals with LTBI infection represent a significant reservoir for new active TB cases (8). Preventive treatment for the LTBI population can achieve a protective effect of 60 to 90% (9). Therefore, identifying individuals at higher risk of developing active TB among LTBI populations is a key strategy for controlling and eliminating TB (10).

Large-scale, population-based LTBI screening remains challenging in resource-limited settings due to concerns about cost-effectiveness and programmatic feasibility (11). However, targeted screening and preventive treatment for high-risk groups, such as close contacts of active TB cases, are widely considered a cost-effective strategy to reduce TB burden. A Chinese cohort study on ATB incidence among rural LTBI individuals found that men and those with a history of TB had progression risks 2.36 times and 5.40 times higher than women and those without a history, respectively (12). Additionally, a study identified that, beyond being male or having a history of PTB, being aged ≥55 years and having a history of exposure to PTB were both risk factors for developing PTB among household contacts. Infants and young children (<5 years old) (13), alcohol abuse, smoking, and low BMI were identified as factors associated with PTB risk.

In addition to conventional regression-based analyses, machine learning has also been applied to identify factors associated with TB and LTBI. A study in Xinjiang using extreme gradient boosting (XGBoost) identified age, BMI, smoking, diabetes, and TB contact history as key factors associated with LTBI (14). In our previous study among close contacts of PTB patients in Henan Province, we developed support vector machine (SVM) to identify factors associated with LTBI, including contact type and residential area (15).

However, most of these studies either focused on general populations or already diagnosed patients. In contrast, the present study specifically targeted close contacts of PTB patients, the priority population for TB preventive therapy, and used routinely available clinical variables (age, gender, BMI, lifestyle factors) to identify factors associated with PTB. Based on these factors, we developed an exploratory nomogram for individualized risk estimation among close contacts. The identified factors and the nomogram may serve as candidate tools for future prospective cohort studies to validate their predictive value for PTB development.

## 2 Materials and methods

### 2.1 Study design and participants

This retrospective case–control study was conducted in six prefecture-level cities in Henan Province (Zhengzhou, Kaifeng, Anyang, Nanyang, Xinyang, and Zhoukou) and their 15 counties and county-level cities in 2024. The PTB group comprised newly diagnosed PTB patients who had received no prior anti-tuberculosis treatment. All PTB patients completed the questionnaire survey at the time of diagnosis, prior to initiation of anti-tuberculosis treatment. The LTBI group comprised close contacts of a separate group of PTB patients treated for more than 3 months (including those from homes, workplaces, schools, and other settings), after ruling out active TB.

### 2.2 Sample size calculation

Sample size was calculated following the 10 Events Per Variable (EPV) principle (16), which requires at least 10 outcome events per independent variable. With 9 variables in the final model, the minimum required PTB cases were 90. Our study met this requirement with 262 PTB cases.

### 2.3 Inclusion and exclusion criteria

The inclusion criteria for the PTB group were as follows: (1) The diagnosis of PTB complied with the health industry standard of the People’s Republic of China (WS288-2017) (17); (2) age ≥18 years; (3) voluntary provision of written informed consent and cooperation with the investigation. The inclusion criteria for the LTBI group were as follows: (1) Purified Protein Derivative Tuberculin Test (PPD) response >10 mm and with no abnormalities observed on chest imaging scan and no clinical evidence of ATB after physician evaluation; (2) age ≥18 years; (3) voluntary provision of written informed consent and cooperation with the investigation. Individuals were excluded from the study if any of the following criteria were met: (1) prior anti-tuberculosis treatment; (2) pregnancy, breastfeeding, or planning for pregnancy; (3) re-treatment for tuberculosis; (4) extrapulmonary tuberculosis or other severe systemic conditions; (5) HIV-positive status; (6) use of glucocorticoids or immunosuppressants within the previous 3 months; (7) mental system disorders; or (8) any other condition deemed by the researcher to be unsuitable for participation.

### 2.4 Data collection

Baseline data collected included age, gender, height, weight, marital status, educational attainment, occupation, labor intensity, place of residence, travel time to medical facilities, household registration type, annual household income, receipt of minimum living allowance, history of chemical vapor exposure, history of exposure to chemical vapours, ventilation status, and disinfection situation. Tuberculosis-related information included recent close contact history with PTB patients, types of contact, duration of contact, receipt of health education, and Bacille Calmette-Guérin (BCG) scar status. Lifestyle and physical condition variables included smoking history, alcohol consumption, abnormal sleep at night, physical exercise status, and self-reported high stress or unstable emotions in the past 6 months. All data were collected by trained staff from designated TB hospitals in the surveyed areas through standardized questionnaire surveys. All investigators received uniform and rigorous training prior to data collection.

### 2.5 Statistical analysis

Participants with incomplete data on any independent variable were excluded from the analysis. This dataset was randomly split into a training set (70%) and a validation set (30%) using the “caret” package in R software. Comparison of baseline characteristics between the training and validation sets revealed no significant differences except for disinfection status (*p* < 0.05), which was therefore excluded from subsequent analyses to avoid potential bias. To avoid data leakage and optimism bias, all variable selection, data preprocessing, and model optimization were performed after the dataset was split into training and validation sets, and exclusively in the training set. The validation set was kept completely blind throughout the model development process and was used solely for the final evaluation of model performance. Categorical variables were expressed as frequencies and percentages. The chi-square test was used to compare baseline characteristics between groups. Multivariable logistic regression analysis was performed to identify factors associated with PTB. The nomogram was built using the “rms” package in R. Model discrimination was assessed using receiver operating characteristic (ROC) curves and the area under the curve (AUC), calculated with the “pROC” package. Internal validation was performed using 1,000 bootstrap resamples, and calibration was assessed using calibration curves generated with the “rms” package. Clinical utility was evaluated using decision curve analysis (DCA), with standardized net benefit calculated using the “rmda” package. All *p*-values were two-sided, and *p* < 0.05 was considered statistically significant.

### 2.6 Ethical approval

This study received ethical approval from the Ethics Committee of Henan Provincial Center for Disease Control and Prevention (Approval No.: 2024-KY-005-02). All participants provided written informed consent after being fully informed of the research background, potential risks and benefits, and the voluntary nature of participation.

## 3 Results

### 3.1 Participant characteristics

A total of 754 participants were included, comprising 365 PTB patients and 389 LTBI individuals. The training set included 528 participants (262 PTB patients) and the validation set included 226 participants (103 PTB patients). The mean age was 51.21 ± 20.02 years in the PTB group and 46.18 ± 15.84 years in the LTBI group, and the proportion of males was 63.7% in the PTB group vs. 39.5% in the LTBI group.

### 3.2 Characteristics selection in the training set

As shown in Table 1, significant differences were observed between the LTBI and PTB groups in the training set for the following variables: age, gender, BMI, marital status, educational attainment, occupation, place of residence, travel time to medical facilities, annual household income, ventilation status, receiving health education, BCG scar status, smoking history, alcohol consumption, physical exercise status, self-reported high stress or emotional instability in the past 6 months, diabetes, and comorbidities (other than diabetes) (*p* < 0.05). No significant differences were found for the remaining variables (*p* > 0.05).

**Table 1:** Results of single-factor analysis for the training set.

Variables	Total number of cases (*n*, %)	LTBI (%)	PTB (%)	*χ* ^2^	*p*
Age				**29.165**	**<0.001**
<60	366 (69.3)	213 (80.1)	153 (58.4)		
≥60	162 (30.7)	53 (19.9)	109 (41.6)		
Gender				31.119	**<0.001**
Female	256 (48.5)	161 (60.5)	95 (36.3)		
Male	272 (51.5)	105 (39.5)	167 (63.7)		
BMI				79.901	**<0.001**
Underweight	76 (14.4)	10 (3.8)	66(25.2)		
Normal	284 (53.8)	134 (50.4)	150 (57.3)		
Overweight	119 (22.5)	81 (30.5)	38 (14.5)		
Obese	49 (9.3)	41 (15.4)	8 (3.1)		
Marital status				18.484	**<0.001**
Unmarried	104 (19.4)	42(15.8)	62 (23.7)		
Married	400 (75.8)	220 (82.7)	180 (68.7)		
Divorced/Widowed	24 (4.5)	4 (1.5)	20 (7.6)		
Academic qualifications				6.143	**0.046**
Primary school and below	171 (32.4)	74 (27.8)	97 (37.0)		
Secondary school/equivalent qualifications	228 (43.2)	118 (44.4)	110 (42.0)		
College and above	129(24.4)	74 (27.8)	55 (21.0)		
Occupation				14.007	**0.003**
Medical personnel	11 (2.1)	3 (1.1)	8 (3.1)		
Migrant workers/farmers/herders	266 (50.4)	116 (43.6)	150 (57.3)		
Students	50 (9.5)	30 (11.3)	20 (7.6)		
Other	201 (38.1)	117 (44.0)	84(32.1)		
Labour intensity				1.005	0.605
Light labour	329 (62.3)	168(63.2)	161 (61.5)		
Moderate labour	186 (35.2)	90(33.8)	96 (36.6)		
Heavy labour	13 (2.5)	8 (3.0)	5 (1.9)		
Place of residence				9.489	**0.002**
Rural	291 (55.1)	129 (48.5)	162 (61.8)		
City	237 (44.9)	137 (51.5)	100 (38.2)		
The time required to reach medical facilities				6.062	**0.048**
<30 min	390(73.9)	207 (77.8)	183 (69.8)		
30-60 min	108 (20.5)	43(16.2)	65 (24.8)		
>60 min	30 (5.7)	16 (6.0)	14 (5.3)		
Type of household registration				0.905	0.341
Local	420 (79.5)	216 (81.2)	204 (77.9)		
Migrant population	108 (20.5)	50 (18.8)	58(22.1)		
Annual household income				6.710	**0.035**
<30,000	275 (52.1)	124 (46.6)	151 (57.6)		
30,000–5,000	164 (31.1)	90 (33.8)	83 (28.2)		
>5,000	89(16.9)	52 (19.5)	42 (14.1)		
Households receiving the minimum subsistence allowance				0.148	0.700
Yes	38(7.2)	18 (6.8)	20 (7.6)		
No	490 (92.8)	248(93.2)	242 (92.4)		
History of exposure to dust				2.544	0.111
Yes	28(5.3)	10 (3.8)	18 (6.9)		
No	500 (94.7)	256(96.2)	244 (93.1)		
History of exposure to chemical vapours				0.311	0.577
Yes	12 (2.3)	7 (2.6)	5 (1.9)		
No	516 (97.7)	259 (97.4)	257 (98.1)		
Ventilation situation				8.472	**0.004**
No	18 (3.4)	3 (1.1)	15 (5.7)		
Have	510 (96.6)	263(98.9)	247 (94.3)		
The situation of receiving health education				21.673	**<0.001**
Yes	237 (44.9)	146 (54.9)	91 (34.7)		
No	291 (55.1)	120 (45.1)	171 (65.3)		
BCG scar				15.288	**<0.001**
Yes	383 (72.5)	213 (80.1)	170 (64.9)		
No	145 (27.5)	53 (19.9)	92 (35.1)		
Smoking history				20.064	**<0.001**
Never smoked	362 (68.6)	201 (75.6)	161(61.5)		
Occasional smoker	11 (2.1)	2 (0.8)	9(3.4)		
Regular smoker	117 (22.2)	54(20.3)	63(24.0)		
Former smoker	38 (7.2)	9 (3.4)	29 (11.1)		
Drinking history				9.146	**0.027**
Never drinks alcohol	383 (72.5)	198 (74.4)	185 (70.6)		
Occasionally drinks alcohol	37 (7.0)	21(7.9)	16(6.1)		
Frequently drinks alcohol	87 (16.5)	43 (16.2)	44 (16.8)		
Used to drink alcohol but has now stopped	21 (4.0)	4 (1.5)	17 (6.5)		
Physical exercise				4.016	**0.045**
Yes	152 (28.8)	87 (32.7)	65 (24.8)		
No	376 (71.2)	179 (67.3)	197 (75.2)		
Staying up late				1.080	0.299
Yes	195 (36.9)	104 (39.1)	91 (34.7)		
No	333 (63.1)	162 (60.9)	171 (65.3)		
The situations of high stress or unstable emotions in the past 6 months				7.528	**0.006**
Yes	113 (21.4)	44 (16.5)	69(26.3)		
No	415(78.6)	222 (83.5)	193 (73.7)		
Diabetes				10.491	**0.001**
Yes	59 (11.2)	18 (6.8)	41(15.6)		
No	469(88.8)	248 (93.2)	221 (84.4)		
Comorbidities (other than diabetes)				0.164	0.686
Yes	123(23.3)	60(22.6)	63(24.0)		
No	405(76.7)	206(77.4)	199(76.0)		

Bold values indicate chi-square (*χ*²) statistics and *P*-values.

The chi-square value and *P*-value for the Age variable should be moved to the same row as Age, aligning them with the variable label.

**Table 2:** Guidance on variable assignment for multivariate regression analysis.

Variables	Assignment specifications
Type	LTBI = 0, PTB = 1
Age	<60 = 0, ≥60 = 1
Gender	Female = 0, male = 1
BMI	Normal = 0, underweight = 1, overweight = 2, obese = 3
Marital status	Unmarried = 0, married = 1, divorced/widowed = 2
Occupation	Medical personnel = 0, migrant workers/farmers/herders = 1, students = 2, other = 3
The situation of receiving health education	No = 0, yes = 1
BCG scar	No = 0, yes = 1
The situations of high stress or unstable emotions in the past 6 months	No = 0, yes = 1
Diabetes	No = 0, yes = 1
Comorbidities (other than diabetes)	No = 0, yes = 1

### 3.3 Multivariable logistic regression analysis

Multivariable logistic regression analysis was performed with group status (LTBI = 0, PTB = 1) as the dependent variable, incorporating all 19 variables that showed significant differences in univariate analysis. Variable assignments for the dependent and independent variables are detailed in Table 2. The results were presented in Table 3.

**Table 3:** Multivariable logistic regression analysis of factors associated with PTB in the training set.

Variables	*β*	SE	Wald	*p*	OR	95% CI for OR
Age						
≥60 vs. <60	0.816	0.258	10.022	0.002	2.261	1.364–3.746
Gender						
Male vs. female	1.277	0.230	30.826	<0.001	3.587	2.285–5.631
BMI						
Normal			52.077	<0.001		
Underweight vs. normal	1.670	0.399	17.553	<0.001	5.312	2.432–11.602
Overweight vs. normal	−0.933	0.270	11.949	<0.001	0.393	0.232–0.668
Obese vs. normal	−2.090	0.475	19.367	<0.001	0.124	0.049–0.314
Marital status						
Unmarried			20.376	<0.001		
Married vs. unmarried	−1.676	0.417	16.148	<0.001	0.187	0.083–0.424
Divorced/widowed vs. unmarried	−0.28	0.72	0.151	0.698	0.756	0.184–3.103
Occupation						
Medical personnel			16.084	<0.001		
Migrant workers/farmers/herders vs. medical personnel	−1.939	0.821	5.577	0.018	0.144	0.029–0.719
Students vs. medical personnel	−3.396	0.931	13.316	<0.001	0.034	0.005–0.208
Other vs. medical personnel	−2.386	0.819	8.484	0.004	0.092	0.018–0.458
The situation of receiving health education						
Yes vs. no	−0.807	0.229	12.388	<0.001	0.446	0.285–0.699
BCG scar						
Yes vs. no	−0.7	0.251	7.745	0.005	0.497	0.303–0.813
The situations of high stress or unstable emotions in the past 6 months						
Yes vs. no	0.622	0.276	5.073	0.024	1.862	1.084–3.199
Diabetes						
Yes vs. no	1.329	0.388	11.749	<0.001	3.779	1.767–8.081
Constant	3.369	0.927	13.207	<0.001	29.041	

The following factors were associated with increased risk of PTB: age ≥60 years (OR = 2.261, 95% CI: 1.364–3.746), male gender (OR = 3.587, 95% CI: 2.285–5.631), diabetes (OR = 3.779, 95% CI: 1.767–8.081), underweight (OR = 5.312, 95% CI: 2.432–11.602), and self-reported high stress (OR = 1.862, 95% CI: 1.084–3.199).

The following factors were associated with decreased risk of PTB: married status (OR = 0.187, 95% CI: 0.083–0.424), migrant workers/farmers/herders (vs. medical personnel, OR = 0.144, 95% CI: 0.029–0.719), students (vs. medical personnel, OR = 0.034, 95% CI: 0.005–0.208), other occupations (vs. medical personnel, OR = 0.092, 95% CI: 0.018–0.458), overweight (OR = 0.393, 95% CI: 0.232–0.668), obesity (vs. normal, OR = 0.124, 95% CI: 0.049–0.314), receipt of health education (OR = 0.446, 95% CI: 0.285–0.699), and presence of a BCG scar (OR = 0.497, 95% CI: 0.303–0.813).

### 3.4 Exploratory nomogram for PTB risk estimation

**Figure 1:**
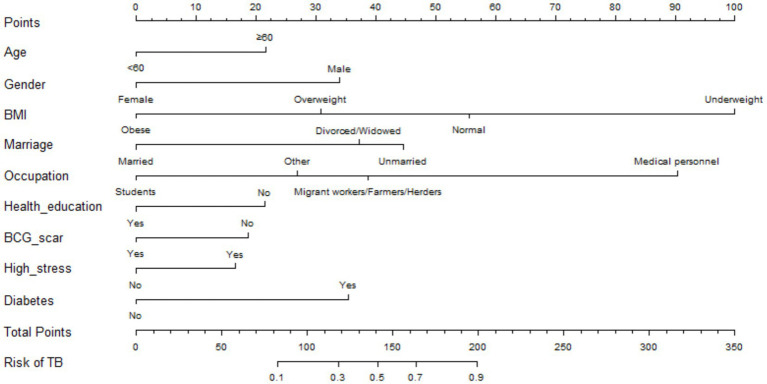
Exploratory nomogram for estimating the probability of PTB among LTBI individuals screened from close contacts.

Based on the multivariable logistic regression results, an exploratory nomogram was developed to visualize the estimated probability of PTB among individuals with LTBI (Figure 1). The nomogram incorporated 9 factors: age, gender, BMI, occupation, marital status, receipt of health education, BCG scar, and self-reported high stress or emotional instability and diabetes. Each factor corresponded to a point score. The total score was calculated by accumulating these scores and the corresponding scores of each factor, summing the points for all factors, and the total score was then mapped to the estimated probability of PTB at the bottom of the nomogram. Example: A 66-year-old male farmer, married, overweight, who had received TB health education, reported no stress or emotional instability in the past 6 months, had a BCG scar, and had diabetes would have a total score of 172 points (22 points for age, 34 points for male gender, 48 points for occupation, 0 points for marital status, 32 points for overweight, 0 points for health education, 0 points for no stress, 0 points for BCG scar, and 36 points for diabetes), corresponding to an estimated probability of PTB of approximately 74%.

### 3.5 Model performance evaluation

**Figure 2:**
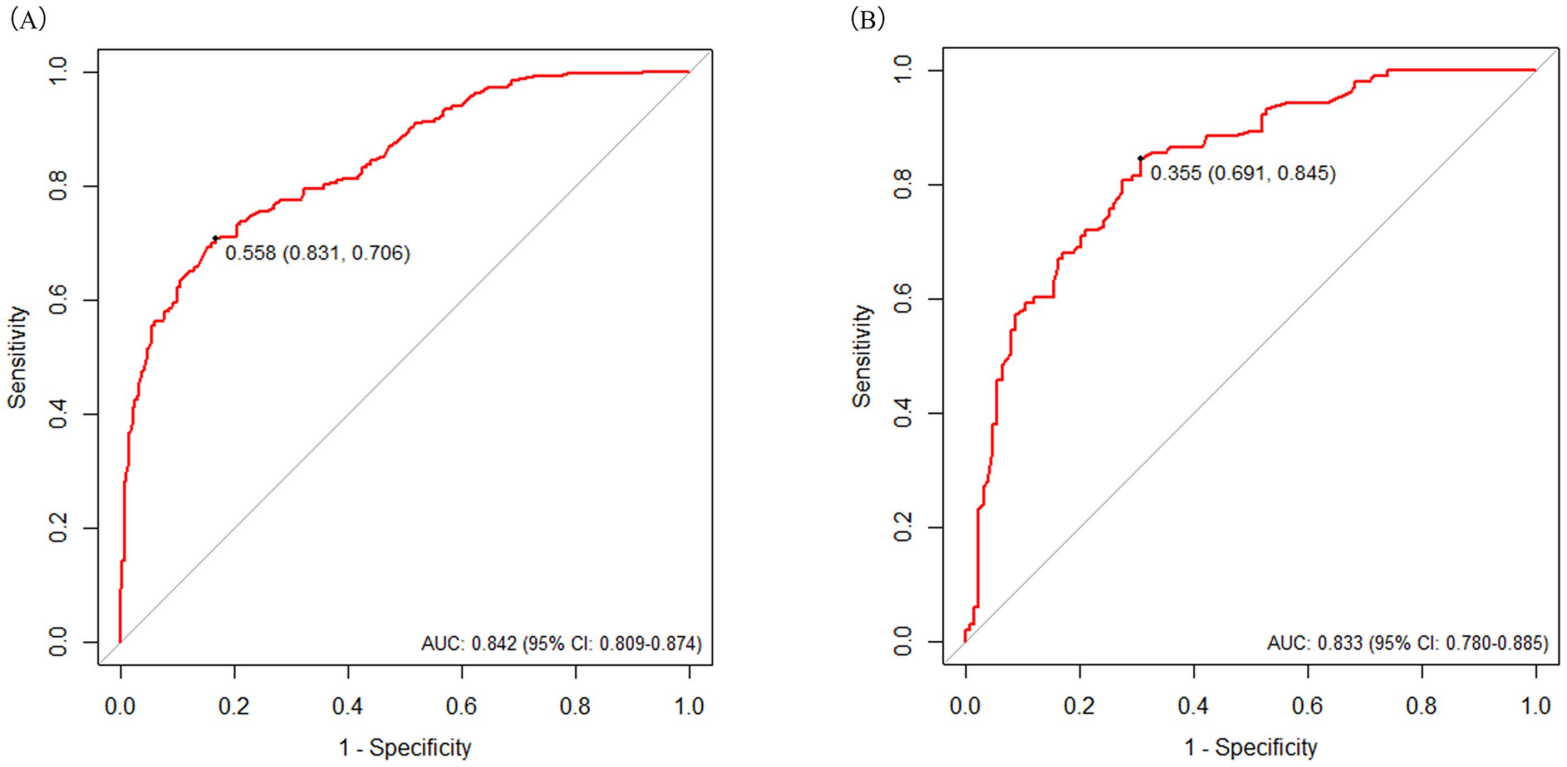
ROC curves of the nomogram for PTB risk estimation in the training **(A)** and validation **(B)** sets.

**Figure 3:**
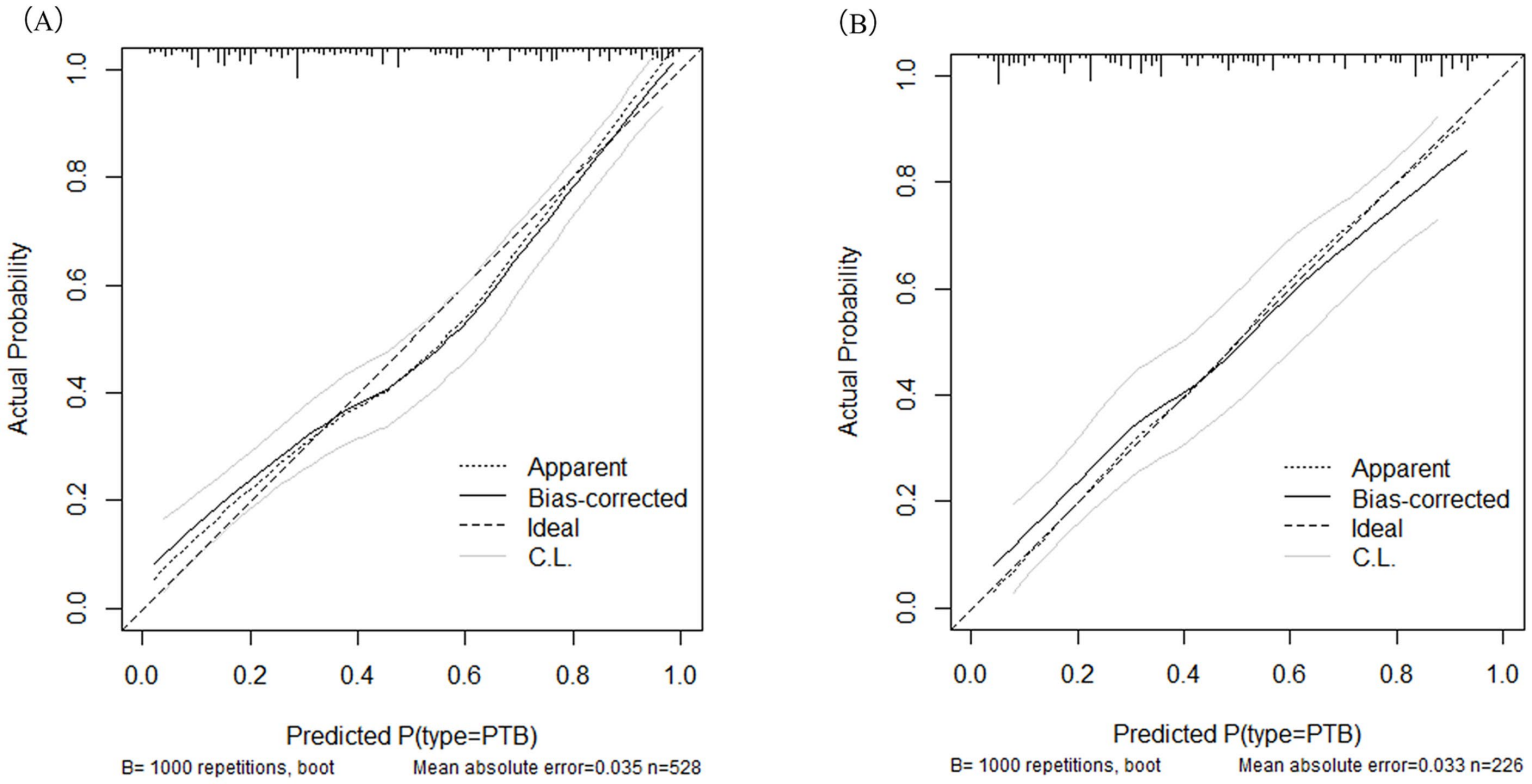
Calibration curves of the exploratory nomogram for PTB risk estimation in the training set **(A)** and validation set **(B)**.

**Figure 4:**
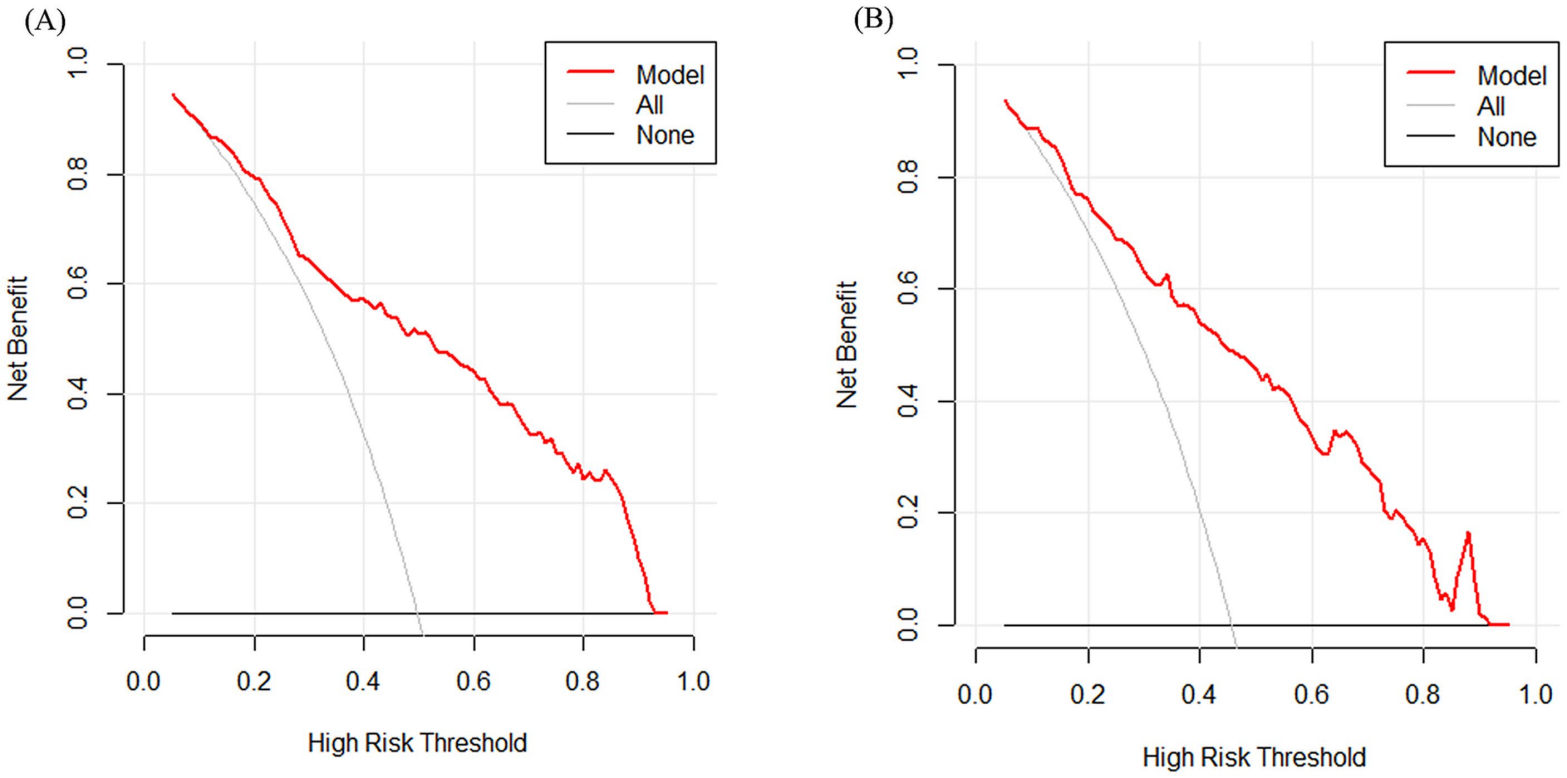
Decision curves of the exploratory nomogram for PTB risk estimation in the training set **(A)** and validation set **(B)**.

The performance of the exploratory nomogram was assessed in terms of discrimination, calibration, and clinical utility. The discriminative ability of the model was evaluated using ROC curves. The AUC was 0.842 (95% CI: 0.809–0.874) in the training set and 0.833 (95% CI: 0.780–0.885) in the validation set (Figures 2A,B), indicating good discriminative ability. Turning to calibration, the calibration curves (Figures 3A,B) and quantitative metrics showed good performance. The mean absolute error was 0.035 in the training set and 0.033 in the validation set. The calibration intercept was 0.003 and −0.0014, and the calibration slope was 0.9832 and 1.0043, respectively, both close to ideal values. The Brier score was 0.1626 in the training set and 0.1646 in the validation set. Together, these results indicated good calibration and no overfitting. Regarding clinical utility, decision curve analysis (Figures 4A,B) demonstrated that the nomogram achieved higher net benefits than default strategies across threshold ranges of 9–92% in the training set and 8–91% in the validation set, suggesting potential clinical utility.

## 4 Discussion

The World Health Organization (WHO) End TB Strategy aims to reduce TB incidence by 90% and mortality by 95% between 2015 and 2035 (18). Individuals with LTBI serve as reservoirs for MTB. As long as this reservoir persists, eradicating TB remains an unattainable goal (18). However, large-scale LTBI screening and treatment are impractical due to diagnostic limitations, risks of side effects, and high costs (10). Therefore, identifying factors associated with PTB among high-risk populations, such as close contacts of PTB patients, is an important step toward informing targeted prevention strategies.

Our study identified 16 factors by comparing differences in demographic characteristics and socioeconomic variables between LTBI group and PTB group. Further multivariate logistic regression analysis confirmed 9 independently associated with PTB: age ≥60 years, male gender, BMI (underweight, overweight, and obesity), marital status, occupation, receipt of health education, BCG scar, self-reported high stress or emotional instability in the past 6 months, and diabetes. Based on these findings, we developed an exploratory nomogram to visualize the estimated probability of PTB among close contacts.

Male gender was associated with increased PTB risk, consistent with a study in rural China which reported that men had 2.36 times higher risk of developing TB than women (11). This association may be explained by several factors. First, men tend to have more frequent social activities, which may increase their risk of MTB exposure. Second, smoking is more common among men. It impairs immune function and weakens respiratory defense mechanisms, which may increase the risk of developing PTB (19). This is supported by our univariate analysis, in which smoking was significantly associated with PTB (*p* < 0.05) (20). Third, men may experience greater psychological and economic pressures that can adversely affect immune function (21). Fourth, emerging evidence suggests that biological differences between sexes may also influence susceptibility to MTB infection and disease development (22).

Being married was associated with lower PTB risk compared with being unmarried, consistent with previous research (23). Married individuals may have better social support systems to improve quality of life, while unmarried status may lead to higher levels of stress, depression, and anxiety, which can weaken immune function and increase susceptibility to TB infection. A large study from Nigeria found no significant association between marital status and TB incidence among HIV-infected adults (24). This inconsistency may reflect differences in social support systems, living conditions, and healthcare access across different cultural and economic contexts.

Diabetes was associated with increased PTB risk among close contacts (OR = 3.779). This finding is consistent with a large meta-analysis of 47 studies (including 23 case–control studies) involving over 3.6 million controls, which reported that diabetes was associated with a 2.4-fold increased risk of active TB among case–control studies (25). The observed association is biologically plausible. Diabetes impairs host immunity through hyperglycemia-induced dysfunction of macrophages, neutrophils, and T cells, reducing phagocytosis and cytokine production, which may facilitate progression from LTBI to active PTB (26). Future prospective studies are needed to confirm this association and to assess whether diabetes management reduces PTB risk in this population.

Self-reported high stress or emotional instability in the past 6 months was associated with increased PTB risk. Mental health is a key determinant of TB risk: a systematic review demonstrated that individuals with psychiatric disorders (e.g., depression, schizophrenia) have significantly higher TB incidence (27). Emerging evidence indicates that chronic stressors and poor mental health directly affect the immune system, including susceptibility to infection (28–31).

Age ≥60 years was associated with increased PTB risk, consistent with previous findings that older adults individuals have higher TB risk (11). This may be explained by age-related decline in immune function, lower health awareness leading to delayed healthcare seeking, and a higher burden of underlying comorbidities, all of which may increase the risk of MTB infection and transmission (32).

Low BMI was associated with increased PTB risk, while overweight and obesity were associated with decreased risk. This is consistent with previous studies showing that TB patients have lower BMI than LTBI controls (33) and each unit decrease in BMI increases the risk of PTB by approximately 14% (34). The relationship between nutrition and TB is bidirectional. TB is a chronic wasting disease that causes reduced appetite and altered metabolic processes, meaning the disease itself can lead to malnutrition (35). Conversely, malnutrition compromises immune responses and increases susceptibility to TB infection (36).

Working as medical personnel was associated with increased TB risk. In countries with a high TB burden, medical personnel had significantly higher odds of TB infection compared to the general population (OR = 2.27, 95% CI: 1.61–3.20), confirming that TB is an important occupational health problem among medical personnel (37, 38). This may be due to a multifactorial combination of occupational exposure risks, work environment factors, biological factors, and psychological stress among medical personnel. These findings highlight the importance of occupational health protection for medical personnel in high-burden settings.

Receipt of TB health education was associated with lower PTB risk. This association may be attributed to the positive effect of health education on tuberculosis knowledge, attitudes, and practices (KAP). Studies among close contacts of TB patients have demonstrated that health education significantly improves KAP levels regarding TB prevention (39, 40). Specifically, individuals who had received TB health education had greater awareness of the disease’s transmission routes, symptoms, and preventive measures, which may motivate them to adopt protective behaviors such as wearing masks correctly, maintaining good ventilation, and avoiding close contact with TB patients, thereby reducing the risk of TB infection and progression.

Presence of a BCG scar was associated with lower PTB risk. BCG is the only currently available vaccine against TB (41). However, a meta-analysis indicated that BCG vaccination at birth was effective in preventing TB in young children but not in adolescents and adults (42), highlighting the urgent need for novel vaccines to improve protection across all age groups.

The exploratory nomogram showed good discriminative ability and calibration in internal validation. Decision curve analysis suggested potential clinical utility pending external validation.

However, this study has the following limitations: First, this was a retrospective case–control study. Although variables were measured at diagnosis, reverse causation cannot be excluded (e.g., subclinical weight loss may have affected BMI); several key variables (e.g., prior TB exposure intensity, immunological status) were not available; we could not distinguish between reactivation and recent primary infection, which is inherent to retrospective studies but does not affect our core conclusions. Thus, these variables are exploratory correlates requiring prospective validation. Second, our LTBI controls were close contacts, a high-risk subgroup rather than the general LTBI population. Therefore, our nomogram applies only to individuals with documented TB exposure. Third, model validation was internal only, lacking independent external validation. Therefore, our nomogram is not yet ready for clinical use and requires external validation in future studies. Fourth, stress was assessed using non-standardized retrospective questions, which may have introduced recall bias; therefore, this finding should be interpreted as exploratory. Fifth, we used the tuberculin ski00n test (TST) alone for LTBI diagnosis. In the BCG-vaccinated Chinese population, TST is associated with false-positive results, and its sensitivity is suboptimal in immunocompromised individuals, potentially leading to underdiagnosis. Future studies combining TST with IGRA are warranted to improve diagnostic accuracy.

## 5 Conclusion

In this retrospective case–control study of close contacts of PTB patients in Henan Province, China, age ≥60 years, male gender, diabetes, underweight, and self-reported high stress were identified as factors associated with increased PTB risk, while married status, certain occupations, overweight, obesity, receipt of TB health education, and presence of a BCG scar were associated with decreased PTB risk. Based on these findings, an exploratory nomogram was developed to visualize the estimated probability of PTB among close contacts. The nomogram demonstrated good discriminative ability and satisfactory calibration in internal validation. The identified factors may serve as candidate variables for future prospective cohort studies to validate their predictive value for PTB development. However, as this was a retrospective study with internal validation only, the nomogram is not yet ready for clinical use and requires external validation in geographically diverse populations before clinical application.

## Data Availability

The original contributions presented in the study are included in the article/Supplementary material, further inquiries can be directed to the corresponding authors.
